# Short communication: evaluation of feeding a glycerol-based electrolyte solution prior to harvest in beef steers

**DOI:** 10.1093/tas/txaf124

**Published:** 2025-09-16

**Authors:** Federico Podversich, Jason E Griffin, Jeff S Heldt, Warren C Rusche, Zachary K Smith

**Affiliations:** Department of Animal Science, South Dakota State University, Brookings, SD 57007, United States; Department of Animal Science, South Dakota State University, Brookings, SD 57007, United States; Selko® USA, Indianapolis, IN 46241, United States; Department of Animal Science, South Dakota State University, Brookings, SD 57007, United States; Department of Animal Science, South Dakota State University, Brookings, SD 57007, United States

**Keywords:** electrolyte solution, glycerol, pre-harvest

## Abstract

The objective of this experiment was to determine if supplementation with a glycerol-based electrolyte solution in the drinking water prior to shipping for harvest increased carcass weight and enhanced carcass quality in beef steers. The solution used (HydraFit—Selko USA, Indianapolis IN) contained: potassium chloride, acetic acid, magnesium hydroxide, sodium propionate, and sodium chloride; 5.10% potassium, 1.00% sodium, and 0.87% magnesium. Steers (*n* = 40; 20 steers per treatment; BW = 659 ± 72.9 kg) were weighed 40 hour prior to harvest, blocked by BW (*n* = 4 BW blocks), and assigned to one of two treatments: no glycerol-based electrolyte solution (Control) or use of a glycerol-based electrolyte solution (GES) added to the water at 4% vol/vol (4 pens/treatment, 8 pens total). Feed and water access was not restricted nor was a shrink percentage applied to any BW measures. Thirty-six hours prior to harvest a 378 L water tank was filled with water from the same source the steers had been consuming for the previous 126 d. Water and feed were introduced 36 hour prior to harvest. Final BW was captured immediately before shipping (12 hour prior to harvest). Cattle were transported 98 km to a commercial abattoir. Cattle were held in lairage with ad libitum access to water prior to harvest the following morning. Water intake pre-shipment was determined after the cattle were removed from the pens and transported to slaughter, allowing 22.5 hours of access to feed and water prior to shipping. Water volume was estimated by measuring water depth remaining in the tank. Depth measurements were calibrated to water volume by metering (DM-P; Assured Automation, Roselle, NJ; ± 1.0%) 0.64 cm of water into each tank and recording the liters of water metered. Hot carcass weight was determined at the time of slaughter and cold carcass weight was determined after a 48-hour chill. Providing a glycerol-based electrolyte solution, Hydrafit, to finished beef cattle in their drinking water for approximately 22.5 hours prior to shipping for harvest tended to increase the final live weight (*P* = 0.08) by 1%, but had no effect (*P *≥ 0.88) on hot or cold carcass weight.

## Teaser

Providing a glycerol-based electrolyte solution in the drinking water of steers before harvest appears to help maintain body weight.

## Introduction

Dehydration leads to detrimental effects on physiological functions, health status, behavior and welfare (Constable 2003). Hydration is important for the maintenance of normal physical and cognitive functions as well as thermoregulation. The rumen serves as a large water reservoir that enables the animal to go without water for days and then rapidly rehydrate without any negative effects (Constable 2003). The ruminal epithelium can absorb large volumes of water quickly, moving across the rumen wall by gradient of osmolarity between the ruminal fluid and blood perfusing the ruminal epithelium (Constable 2003).

The use of electrolyte supplements in drinking water, has been investigated to attenuate the effects of transport stress on beef cattle (Schaefer et al. 1997; Beatty et al. 2007; Arp et al. 2011). Potassium and magnesium are primary intracellular cations and sodium is primary an extracellular cation (NASEM 2016). These three minerals aid in the maintenance of cellular water balance. Additionally, muscle cells can contain up to 1% of their weight as glycogen (Reece et al., 2015). Pre-slaughter stress can lead to depletion of muscle glycogen, resulting in increased postmortem muscle pH, less desirable meat color, and increased water-holding capacity (Schaefer et al.1990; Arp et al. 2011). Glycerol is an osmolyte and a glucogenic precursor in ruminants that is primarily fermented to propionate in the rumen (Long et al. 2015). However, a proportion of glycerol is not fermented to propionate by ruminal bacterial species but rather absorbed via passive diffusion across the ruminal epithelium (Werner Omazic et al. 2013; 2015). Wilms et al. (2023), observed a linear increment in blood glucose of cattle deprived of feed for 24 and 48 hours when increased glycerol was provided in the drinking water.

Pre-slaughter electrolyte supplementation of fed cattle improves animal hydration, facilitating postmortem pH decline and improved beef color (Schaefer et al. 2001; 2006; Arp et al. 2011). In theory, supplementation of glycerol-based electrolyte solutions can provide simultaneously hydration and a source of glucose for the muscles. Previous studies suggest that a glycerol-based electrolyte solution can be useful under periods of limited access to feed and water, after parturition, or following extreme heat events (Arp et al. 2011; Werner Omazic et al. 2013; Egea et al. 2015). However, to our knowledge no research has evaluated the influence of a glycerol-based electrolyte solution to steers under minimal transit or environmental stress prior to harvest.

The objective of this experiment was to determine the effects of the application in the drinking water of a glycerol-based electrolyte solution (glycerol-based electrolyte solution containing: potassium chloride, acetic acid, magnesium hydroxide, sodium propionate, and sodium chloride; 5.10% potassium, 1.00% sodium, and 0.87% magnesium; HydraFit—Selko USA, Indianapolis IN) prior to shipping for harvest on water consumption and cold carcass weight in beef steers. We hypothesized that the supplementation with the electrolyte solution would increase water consumption and cold carcass weight and would reduce the carcass shrink.

## Materials and methods

The animal care and handling protocols used in this study were approved by the South Dakota State University Institutional Animal Care and Use Committee #2201-001E. This study was conducted at the Southeast Research Farm Feedlot (SERF) located near Beresford, South Dakota, on February 7 and 8 of 2022.

Prior to enrolling in this study, the steers were fed for 126 d and had received a high concentrate finishing diet for the previous 105 d (Table 1). Ractopamine HCl was included in the diet to all pens, for an intended intake of 300 mg per steer daily for the final 28 d on feed. Forty crossbred beef steers were weighed and blocked by body weight [(BW); *n* = 4 blocks]. Within blocks, pens were assigned to one of two treatments (8 pens total; n = 4 pens/treatment). The treatments consisted of (i) Control: no supplementation with the electrolyte solution in the drinking water (CTL), and (ii) inclusion of the electrolyte solution (HydraFit Selko USA, Indianapolis IN) at 4% vol/vol in the drinking water for 22.5 hour prior to shipping to harvest (GES).

**Table 1.** Composition of the finishing diet fed to the steers.

Item	
***Ingredients inclusion, % on dry matter basis***	
**Dry-rolled corn**	56.00
**Modified distillers’ grains plus solubles**	20.00
**Whole-plant corn silage**	20.00
**Molasses-based liquid supplement^a^**	4.00
***Analyzed nutrient composition***	
**Crude protein, %**	14.90
**Non-protein nitrogen^b^, %**	1.35
**Acid detergent fiber, %**	11.35
**Neutral detergent fiber, %**	22.80
**Net energy for gain, Mcal/kg**	1.36
**Calcium, %**	0.66
**Magnesium, %**	0.25
**Phosphorus, %**	0.46
**Potassium, %**	0.96
**Sulfur, %**	0.2
**Cobalt, mg/kg**	0.5
**Copper, mg/kg**	12.0
**Iron, mg/kg**	109.0
**Manganese, mg/kg**	40.0
**Selenium, mg/kg**	0.5
**Zinc, mg/kg**	106.0

a Contained on a DM basis: 30,844 IU of Vitamin A/kg, 170 IU of Vitamin E/kg, 827 g of monensin/metric ton (Elanco Animal Health, Greenfield, IN), and 165 g of tylosin/metric ton (Elanco Animal Health).

b Non-protein nitrogen, CP equivalent.

All steer received 300 mg/steer daily ractopamine hydrochloride for the 28 d prior to harvest.

Steers were housed in 12.15  × 4.5 m partially covered concrete-floor pens (5 steers/pen) with concrete bunks (4.5 m linear bunk space). Each pen was equipped with a 378 L water tank in addition to an automatic waterer. Thirty-six hours prior to harvest, the automatic water fountain in each pen was turned off and the water tanks were filled with the same water source supplying the automatic water fountains.

Water samples were obtained for chemical analysis (Table 2; ServiTech Laboratories, Hastings, NE). Water intake was determined after the cattle were removed from the pens and transported to slaughter. Because the tanks were not completely symmetrical, water volume for every 0.64 cm on a wooden meter stick was correlated with the amount of water remaining in the tank. This calibration was accomplished by using flow meters with an accuracy of ± 1.0% (DM-P Series Multi-purpose Flow Meters; Assured Automation; Roselle, NJ). For each tank, 0.64 cm of water was added with the metered liters of water recorded.

**Table 2.** Chemical composition^a^ of basal water supplied to the steers.

Item	Result
**pH**	8.37
**Chloride, mg/L**	19.1
**Total Hardness, mg/L**	170.0
**Nitrate-Nitrogen, mg/L**	0.6
**Calcium, mg/L**	40.0
**Magnesium, mg/L**	18.0
**Phosphorous, mg/L**	< 0.5
**Potassium, mg/L**	7.0
**Sodium, mg/L**	84.5
**Sulfate, mg/L**	240.0
**Aluminum, mg/L**	0.07
**Cobalt, mg/L**	<0.005
**Copper, µg/L**	10.0
**Iron, mg/L**	0.09
**Manganese, mg/L**	0.017
**Molybdenum, µg/L**	10.0
**Selenium, µg/L**	5.0
**Total dissolved solids, mg/L**	448.0

a Analyzed by ServiTech Laboratories, Hastings, NE.

Initial BW was captured 40 hour prior to harvest, and no feed or water was withheld prior to BW measurement. No pencil shrink was applied to any BW measures. Water and feed were introduced at 22.5 hour prior to shipping (36 hour prior to harvest). Final BW was captured 12 hour prior to harvest and immediately before shipping.

Cattle were loaded onto trucks and transported 98 km to a commercial abattoir. Cattle were held in lairage without feed, for ∼12 hours, but with ad libitum access to water (without any solution added for any of the groups). Electronic ID tags were used to determine harvest order. Steers were harvested the following morning at 0900 hour. At the commercial abattoir, steers were randomly presented for slaughter using standard U.S. beef industry practices and USDA/Food safety inspection service criteria. Hot carcass weight (HCW) was determined at the time of slaughter and cold carcass weight (CCW) was determined after a 48-hour chill. One steer from Con was condemned at the abattoir for reasons not related to treatment, this steer’s contribution to the pen mean was deleted from the entire data set.

Dressing percentage (DP) was calculated for both HCW (HCW-DP) and and CCW. Carcass shrink was calculated as the percentage of shrink from HCW to CCW, with the formula: [(1- (CCW/HCW)) ×100]. Rib eye area (REA), rib fat (RF), marbling score, kidney-pelvic-heart fat (KPH), and Yield Grade were determined from video recordings based on United States Department of Agriculture (USDA 2017). Liver abscess prevalence was not considered for the current study, since the development of an abscess takes between 3 to 10 days, after the coagulative necrosis (Amachadi and Nagaraja 2022). Therefore, it is unlikely that the electrolyte supplementation influenced liver abscess development.

### Statistical analysis

All data were analyzed as a randomized complete block design using the GLIMIX procedure of SAS 9.4 (SAS Inst. Inc., Cary, NC) with pen as the experimental unit. The model included the fixed effect of dietary treatment, and the random effect of block. Least squares means were generated using the LSMEANS statement of SAS 9.4. An α of 0.05 was used to determine significance, and an α of 0.06 to 0.10 was considered a tendency.

## Results

Results are presented in Table 3. Providing GES in drinking water for approximately 22 hour prior to shipping for harvest tended to increase final live weight (*P *= 0.08) by 1% (632.7 vs 639.0 kg, for CTL vs. GES, respectively). However, no differences were observed (*P *≥ 0.88) for either HCW or CCW. Also, no differences were observed in water intake (liquid water, feed water, and total water) between treatment groups (*P *≥ 0.18). Similarly, no differences were observed for DP, carcass shrink, REA, RF, nor yield grade between treatments (*P *≥ 0.22). Providing GES in drinking water tended to reduce marbling (*P *= 0.09) and KPH (*P *= 0.08).

**Table 3.** Effect of a glycerol-based electrolyte solution inclusion in drinking water the day prior to shipping for slaughter on water consumption and carcass characteristics of feedlot steers.^a^

	Treatment^a^			
Item	CTL	GES	SEM^b^	*P*-value
**Pens (Steers)**	4 (19)	4 (20)	—	—
***Live traits* ^c^**				
**Initial body weight (BW), kg**	658.5	659.9	1.18	0.29
**Final BW, kg**	632.7	639.0	2.40	0.08
**Water intake, L**	21.9	24.9	2.38	0.30
**Calculated GES intake^d^, L**	—	1.00	—	—
**Dry-matter intake, kg**	7.1	7.9	0.64	0.31
**Feed water intake, L**	4.5	5.0	0.44	0.36
**Total water intake, L**	26.4	29.9	1.97	0.18
***Carcass traits***				
**Hot carcass weight, kg**	404	405	2.8	0.88
**Cold carcass weight, kg**	402	402	2.7	0.97
**Hot carcass dressing percentage^e^, %**	63.9	63.3	0.60	0.36
**Cold carcass dressing percentage^f^, %**	63.6	62.8	0.62	0.35
**Carcass shrink^g^, %**	0.61	0.73	0.219	0.62
**Ribeye area, cm2**	88.5	90.7	3.08	0.51
**Rib fat, cm**	1.30	1.17	0.086	0.22
**Marbling^h^**	481	419	24.6	0.09
**Kidney, pelvic, and heart fat, %**	1.78	1.76	0.008	0.08
**Yield grade**	3.14	2.89	0.198	0.30

a CTL: Control (no electrolyte supplementation), GES: Glycerol-based electrolyte solution (potassium chloride, acetic acid, magnesium hydroxide, sodium propionate, and sodium chloride; 5.10% potassium, 1.00% sodium, and 0.87% magnesium; HydraFit—Selko USA, Indianapolis IN).

b Standard error of the mean (*n* = 4 pens/treatment).

c No shrink was applied to any BW measures. Initial BW was captured on 2/6/22 at 1345 hour, no feed or water was withheld prior to BW measure captured on 2/6/22. Water and feed were introduced at 1825 hour on 2/6/22. Final BW was captured on 2/7/22 at 1630 hour. Intake and water consumption were measured for 22.5 hours. Cattle were loaded onto trucks at 1730 h and shipped 98 km to a commercial abattoir. Cattle were held in lairage with ad libitum access to water and were harvested on 2/8/22 at 0900 hour. Cold carcass weight was captured at 48 hour post-exsanguination.

d Dose: 2.1 L of HydraFit per 51 L of water.

e Calculated as: (HCW/Final BW)×100.

f Calculated as: (CCW/Final BW)×100.

g Calculated as: (1-(CCW/HCW))×100.

h 400 = small^00^.

## Discussion

Glycerol is often fermented by ruminal bacteria to propionate in the rumen (Hales et al. 2015; Long et al. 2015). However, not all glycerol provided to the ruminal environment is fermented to volatile fatty acids. When dairy calves were administered an oral rehydration solution containing glycerol and glucose, plasma glycerol levels were increased with no effect on ruminal VFA production (Werner Omazic et al. 2015). In that study, some of the glycerol was absorbed directly, and only a proportion was utilized by ruminal microorganisms (Werner Omazic et al. 2015). Wilms et al. (2023) observed a linear and a quadratic increase in blood glucose at 24 and 48 hours of feed restriction, respectively, in Holstein bulls when glycerol was added to drinking water. Yet, the authors of that study acknowledge that glycerol may have been transformed to propionate in the rumen, and that compound may later go through gluconeogenesis in the liver (Wilms et al. 2023). In the same study, increased blood glucose was accompanied by a linear reduction in the serum urea-to-creatinine ratio with increased glycerol supplementation, suggesting a decrease in muscle protein catabolism (Wilms et al. 2023).

Egea et al. (2015) supplemented similar levels of glycerol as the present experiment in either a solution or as a drench to beef bulls prior to harvest. The tendency for a greater final BW of steers supplemented with GES in the present study contrasts with the findings by Egea et al. (2015), where no differences were observed for final live weight between bulls supplemented with glycerol or not prior to slaughter. Our findings can be compared to a series of studies by Wilms et al. (2021). In a first study by Wilms et al. (2021), increasing the dose of electrolytes in drinking water linearly increased final BW and reduced BW loss of feed-deprived bulls. However, in another study by Wilms et al. (2021), increasing glycerol levels in the drinking water that already contained electrolytes tended to increase fluid intake without effects on final BW or BW loss. Comparably, Schaefer et al. (1990) observed that bulls offered drinking water containing either dextrose or electrolytes during lairage maintained BW pre-harvesting at 100% of their pre-shipping BW (captured at the farm). In contrast, when only water or no water were offered, pre-harvesting BW was 95% of the pre-shipping BW (Schaefer et al. 1990). Such differences were also expressed in carcass weights (Schaefer et al. 1990). A possible explanation for the marginal effects observed in the current study could be the short time during which the treatments were applied, only one day. Considering the product evaluated uses glycerol as a carrier (minimum 40% glycerol, and 60% DM), the estimated energy contribution of 1 L of the product is 0.36 Mcal Neg (based on Preston 2017). A previous study conducted in Australia with cattle being shipped overseas observed an increase of approximately 3% in BW with 18 d of electrolyte supplementation in the drinking water (Beatty et al. 2007).

The lack of differences observed for carcass weight (hot and cold), carcass shrink, and dressing percentage in our study, aligns with the lack of differences in the study by Egea et al. (2015), where glycerol supplementation did not modify carcass weight, dressing percentage, carcass quality or carcass pH. However, in their study, steaks from bulls supplemented with glycerol showed a 4.1% greater water-holding capacity and a tendency towards reduced cooking yield loss (Egea et al. 2015). In the current study, meat quality attributes were not evaluated, other than collecting carcass measurements. Another consideration is that in the current experiment, cattle were not deprived of feed during the time that GES was offered, as compared to other studies which saw a greater impact of the supplemental solutions (Schaefer et al. 1990; Wilms et al. 2021).

Because of the short length of exposure to GES treatment prior to slaughter (36 hour), it seems unlikely that the tendencies observed for KPH and marbling score are biologically related to the treatments. Similarly, liver abscess prevalence (not considered in this trial) cannot be related to treatments since abscess formation requires a minimum of 3 d after coagulative necrosis is established (Amachadi and Nagaraja 2022).

## Conclusion

Providing a glycerol-based electrolyte solution to finished beef steers 22.5 hour prior to shipping tended to increase final BW. Yet, no differences were observed in carcass-related parameters. Future research should look at timing of application (ie in lairage at the abattoir, a bolus dose prior to shipping, or for some extended period prior to shipping) as a means to minimize weight loss during transport to harvest.

## Data Availability

Data can be made available upon reasonable request to Z.K.S.

## References

[txaf124-B1] Amachawadi R. G. , Nagaraja T. G. 2022. Pathogenesis of liver abscesses in cattle. Vet. Clin. North Am. Food Anim. Pract. 38:335–346. 10.1016/j.cvfa.2022.08.001. 36243456

[txaf124-B2] Arp T. S. et al 2011. Effects of preslaughter electrolyte supplementation on the hydration and meat quality of cull dairy cows. Prof. Anim. Sci. 27:43–51. 10.15232/S1080-7446(15)30443-5.

[txaf124-B3] Beatty D. T. , Barnes A., Taplin R., McCarthy M., Maloney S. K. 2007. Electrolyte supplementation of live export cattle to the Middle east. Aust. J. Exp. Agric. 47:119–124. 10.1071/EA06041.

[txaf124-B4] Constable P. 2003. Fluid and electrolyte therapy in ruminants. Vet. Clin. North Am. Food Anim. Pract. 19:557–597. 10.1016/s0749-0720(03)00054-9 14608802

[txaf124-B5] Egea M. , Linares M. B., Hernández F., Madrid J., Garrido M. D. 2015. Pre-slaughter administration of glycerol as carbohydrate precursor and osmotic agent to improve carcass and beef quality. Livest. Sci. 182:1–7. 10.1016/j.livsci.2015.10.011.

[txaf124-B7] Hales K. E. , Foote A. P., Brown-Brandl T. M., Freetly H. C. 2015. Effects of dietary glycerin inclusion at 0, 5, 10, and 15 percent of dry matter on energy metabolism and nutrient balance in finishing beef steers1. J. Anim. Sci. 93:348–356. 10.2527/jas.2014-8075. 25412753

[txaf124-B8] Long C. J. , Sneed A. D., Schroeder A. R., Felix T. L. 2015. Effects of dietary glycerin on growth performance, carcass characteristics, and rumen metabolism of beef. Prof. Anim. Sci. 31:568–576. 10.15232/pas.2015-01426.

[txaf124-B9] NASEM. 2016. Nutrient requirements of beef cattle. 8th ed. Natl. Acad. Press, Washington, DC.

[txaf124-B10] Preston R. L. 2017. Feed composition table. https://www.beefmagazine.com/cattle-nutrition/feed-composition-tables-data-to-mix-the-best-feed-for-your-cattle

[txaf124-B11] Reece W. O. , Erickson H. H., Goff J. P., Uemura E. E. 2015. Dukes’ physiology of domestic animals. John Wiley & Sons. https://ebookcentral.proquest.com/lib/pit/detail.action? docID=7103738

[txaf124-B12] Schaefer A. L. , Dubeski P. L., Aalhus J. L., Tong A. K. W. 2001. Role of nutrition in reducing antemortem stress and meat quality aberrations. J. Anim. Sci. 79:E91. 10.2527/jas2001.79E-SupplE91x.

[txaf124-B13] Schaefer A. L. et al 2006. The impact of antemortem nutrition in beef cattle on carcass yield and quality grade. Can. J. Anim. Sci. 86:317–323. 10.4141/A05-053.

[txaf124-B14] Schaefer A. L. , Jones S. D., Stanley R. W. 1997. The use of electrolyte solutions for reducing transport stress. J. Anim. Sci. 75:258–265. 10.2527/1997.751258x. 9027574

[txaf124-B15] Schaefer A. L. , Jones S. D. M., Tong A. K. W., Young B. A. 1990. Effects of transport and electrolyte supplementation on ion concentrations, carcass yield and quality in bulls. Can. J. Anim. Sci. 70:107–119. 10.4141/cjas90-012.

[txaf124-B16] United States Department of Agriculture (USDA). 2017. United States standards for grades of carcass beef. Agricultural marketing service, USDA, Washington, DC.

[txaf124-B17] Werner Omazic A. , Kronqvist C., Zhongyan L., Martens H., Holtenius K. 2015. The fate of glycerol entering the rumen of dairy cows and sheep. J. Anim. Physiol. Anim. Nutr. (Berl). 99:258–264. 10.1111/jpn.12245. 25250664

[txaf124-B18] Werner Omazic A. , Tråvén M., Roos S., Mellgren E., Holtenius K. 2013. Oral rehydration solution with glycerol to dairy calves: effects on fluid balance, metabolism, and intestinal microbiota. Acta Agric. Scand. A Ani. Sci. 63:47–56. 10.1080/09064702.2013.785585.

[txaf124-B19] Wilms J. N. , Carvalho I. P., van Empel M., Martín-Tereso J. 2023. Mineral and glycerol concentrations in drinking water on body weight loss and acid-base balance in feed-deprived holstein bulls. J. Anim. Physiol. Anim. Nutr. (Berl). 107:77–88. 10.1111/jpn.13700. 35343631 PMC10084004

